# Income-Related Peripheral Artery Disease Treatment: A Nation-Wide Analysis from 2009–2018

**DOI:** 10.3390/jcdd9110392

**Published:** 2022-11-14

**Authors:** Daniel Messiha, Olga Petrikhovich, Julia Lortz, Amir Abbas Mahabadi, Ramona Hering, Mandy Schulz, Tienush Rassaf, Christos Rammos

**Affiliations:** 1 Department of Cardiology and Vascular Medicine, West German Heart and Vascular Centre, University of Duisburg-Essen, 45147 Essen, Germany; 2 Department of Data Science and Healthcare Analyses, Central Research Institute for Ambulatory Healthcare in Germany (Zi), 10587 Berlin, Germany

**Keywords:** PAD, atherosclerosis, health economy

## Abstract

Economic status has a measurable and significant effect on cardiovascular health. Socioeconomic- and income-related disparities worsen cardiovascular risk factors. Peripheral artery disease (PAD) remains a major risk factor for morbidity and mortality. Not all patients benefit equally from recent advances in outpatient healthcare. The implementation of guideline recommendations regarding treatment is inadequate. Income-related disparities for PAD treatment are unknown. We aimed to analyse income-stratified PAD prevalence, outpatient treatment and pharmacotherapy. Associations of statutory health insurance physicians at the regional level, income-stratified PAD prevalence and differences in outpatient care and pharmacotherapy were analysed in 70.1 million statutorily insured patients/year between 2009 and 2018. Analysis was based on claims data (§295 of the social code (SGB V)) and drug-prescription data (§300 SGB V). The diagnosis of PAD was defined by ICD I70.2-9. Regional income data were derived from the German Census Bureau. PAD prevalence was higher in low-income than in high-income areas. Low-income patients more often presented to angiology outpatient care and more frequently received guideline recommended pharmacotherapy. High-income patients more often presented to outpatient vascular surgery. This was true for statins, antiplatelets, intermittent claudication and critical limb ischemia alike. These data indicate that PAD and income are associated. Regional income is related to insufficiencies in guideline-recommended treatment and contact to vascular specialists. Our results aim to encourage medical professionals to implement PAD guideline recommendations, especially in high-income areas. Further studies on associations between spatial-level income and healthcare in PAD are needed.

## 1. Introduction

Healthcare systems have made advances in recent years, yet not all regions have benefitted equally. Socioeconomic and income-related disparities remain one of the most fundamental determinants regarding cardiovascular health [1,2]. Thus, health and income remain unequally distributed [1,2,3]. Correspondingly, the prevalence of cardiovascular risk factors such as diabetes, obesity, smoking or lack of physical activity have increased the most in low-income cohorts, placing patients from low-income areas at a particularly high risk for cardiovascular events [1,4]. One of the leading causes of mortality and morbidity in the industrialized world is cardiovascular diseases, such as peripheral artery disease (PAD) and coronary artery disease (CAD) [5,6,7].

Worldwide, more than 200 million people suffer from PAD, with approximately 10% of those affected being older than 50 years in Western Europe and North America [8]. All major societies recommend lipid lowering and antiplatelet therapy as the most important tools in reducing the burden of cardiovascular disease [9,10,11]. Despite clear guideline recommendations, implementation is inadequate [12,13].

PAD, characterized in general by a reduced pain-free walking distance and subsequently intermittent claudication (IC), is a disabling condition. The severest stage of PAD, chronic limb-threatening ischemia (CLTI), requires even more attention. According to the Inter-Society Consensus for the Management of Peripheral Arterial Disease (TASC II), CLTI is defined as chronic ischemic rest pain, ulceration or gangrene due to PAD [14]. CLTI is associated with an increased mortality, a high risk of major amputation and impaired quality of life [15,16,17,18]. Frequent complications include infections and gangrene, as well as major amputations. Moreover, CLTI often appears in conjunction with CAD. Those factors directly translate into a severe five-year mortality rate in CLTI, which has been described as being as high as 50% [19,20].

Implementation of guideline recommendations depends on various factors, including income structures, gender, social status and race [21,22]. We recently reported an underuse of guideline-recommended therapy for statutorily insured PAD patients in Germany with IC and CLTI, highlighting the presence of a gender gap in outpatient care [12,13].

Income and dependent social structures, however, are the most fundamental determinants of health status and one of the strongest predictors for contact with outpatient healthcare [23,24,25,26]. Although advances in outpatient healthcare structures have been made in the past decades, income disparities have continuously increased. This might lead to an unequal contact with healthcare providers [2,25,26,27,28].

It is still unclear to what extent income disparities within a progressive European healthcare system influence guideline-recommended outpatient management and pharmacotherapy. To this aim, this study investigated income-based differences in prevalence, outpatient contacts and prescription patterns of PAD stratified by IC and CLTI from 2009 to 2018.

## 2. Materials and Methods

Ambulatory claims data for all statutorily insured patients, comprising 70.1 million patients per year and 87% of the German population, were analysed. A total of 13% of the German population are privately insured and were excluded from the study due to lack of availability of data. Prevalence was stratified by federal regional income within the study frame from 2009 to 2018. This study is based on the ambulatory claims data (2009–2018) of the panel doctors’ services according to §295 SGB V and drug-prescription data (2009–2016) according to §300 SGB V. Diagnosis of PAD was defined according to medical diagnoses of PAD ICD I70.2-9. For the overall analysis of prevalence and outpatient-care distribution of patients with PAD, all patients aged ≥40 years with one of the ICD diagnostic codes consistent with PAD (I70.2, I70.8, I70.9) were included. For the stage related analysis and regarding antiplatelet and lipid-lowering therapy, all ambulatory patients were included regardless of age, if they had one of the following ICD diagnoses codes consistent with PAD: I70.20, I70.21, I70.22, I70.23, I70.24, I70.25, I70.29. For PAD staging, the most severe ICD diagnosis code per patient was used. These diagnostic codes correspond to Fontaine PAD stages I–IV, IC (I70.21, I70.22) and CLTI (I70.23, I70.24, I70.25), respectively. In the case of repetitive ICD diagnoses per patient and year, the most severe diagnosis was included in the analysis.

Special focus was put on guideline adherence to medical treatment with lipid-lowering agents and antiplatelet drugs stratified by IC and CLTI. Income was stratified into low-income and high-income areas according to the monthly per capita gross income for each German federal state in 2021 [29]. Specialized vascular density per 1 million persons was based on register data from the association of statutory health-insurance physicians.

In addition, identification of the current income-based distribution of patients to ambulatory care by the doctors’ specialities (internal medicine, cardiologists, angiologists, vascular surgeons) was based on “lifelong physician codes” (LANR). Potential duplicate counts were not excluded, thus the present data present actual outpatient contacts for each specialty.

Prescription of PAD-related medications was analysed by the “Anatomical Therapeutic Chemical” (ATC) codes indicating that a PAD-related medication had been prescribed from the database in a timeframe from 2009 to 2016. In this study, PAD-related medications included C10AA (statins, HMG CoA reductase inhibitors) and B01AC (platelet aggregation inhibitors, excluding heparin). To count a patient’s consultation to specialized outpatient care or a drug prescription, at least one visit or prescription per year had to be registered in the database per patient. Visits to more than one source of specialized outpatient care were counted multiple times. Individual national doctor’s registration numbers and pharmacotherapy prescription coding made it possible to couple outpatient consultations with prescription rates of pharmacotherapy. The analysis of prevalence and outpatient care distribution was based on 17,633,970 patients from the outpatient claims data. The analysis of PAD stage and pharmacotherapy was based on 4,515,577 patients, derived from pharmacotherapy claims data. Potential duplicate counts between both claim data pools were not excluded. To further evaluate treatment structures, we elucidated the density of specialized vascular care per population count. All analyses were performed with GraphPad Prism 8.0. Statistical analysis was performed with two way ANOVA.

## 3. Results

**Figure 1:**
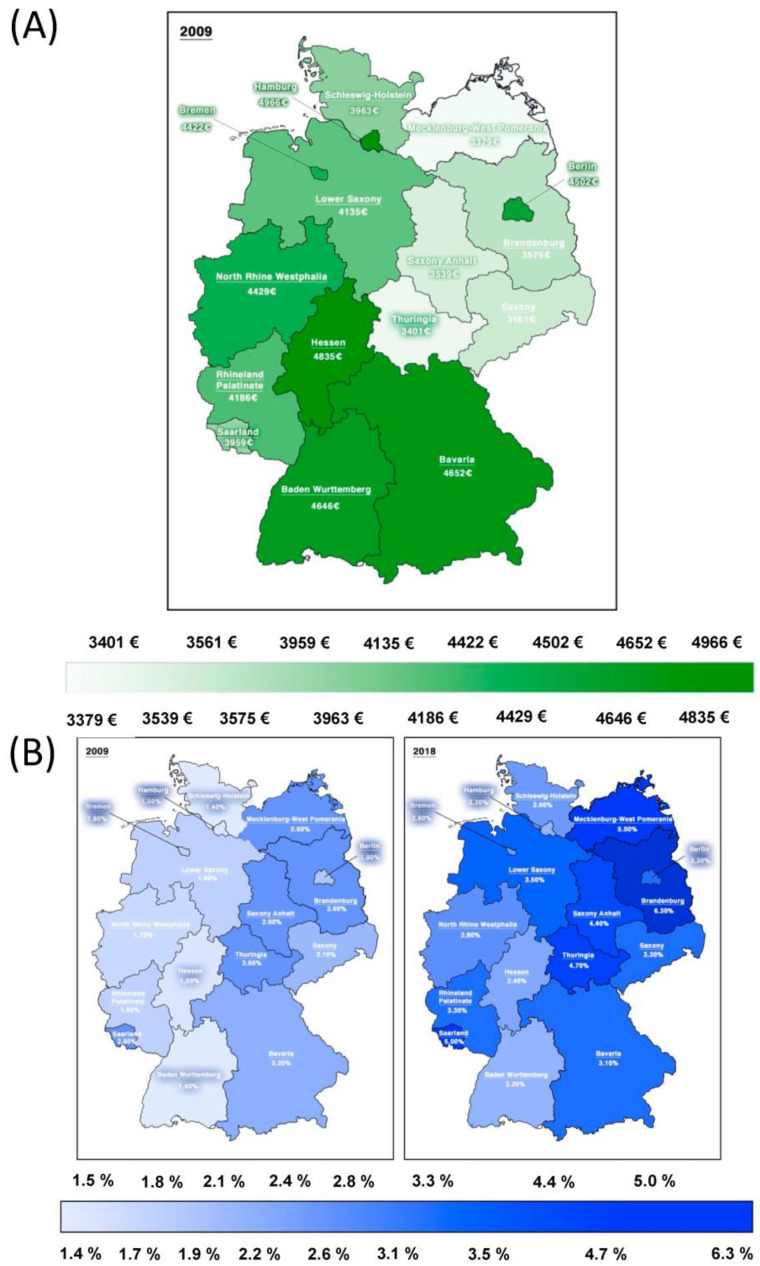
Stratification of German federal states according to income structure in 2009 (**A**) and prevalence of PAD 2009 vs. 2018 (**B**). For analysis of outpatient-care distribution, 17,633,970 patients with PAD were included between 2009 and 2018; 47% were living in low-income areas (average income in low-income areas EUR 3749; in high-income areas EUR 4660).

This study was based on nationwide ambulatory claims data covering approximately 87% of the German population. A total of 70.1 million statutorily insured patients per year were identified. Overall, for the analysis of outpatient-care distribution, 17,633,970 patients with PAD were included between 2009 and 2018, of whom 47% were living in low-income areas (average income per month in low-income areas EUR 3749; in high-income areas EUR 4660) (Figure 1A). For the analysis of pharmacotherapy, 12,900,050 patients between 2009 and 2016 were found to be eligible, of whom 8,134,666 were excluded because of missing PAD-stage status.

**Figure 2:**
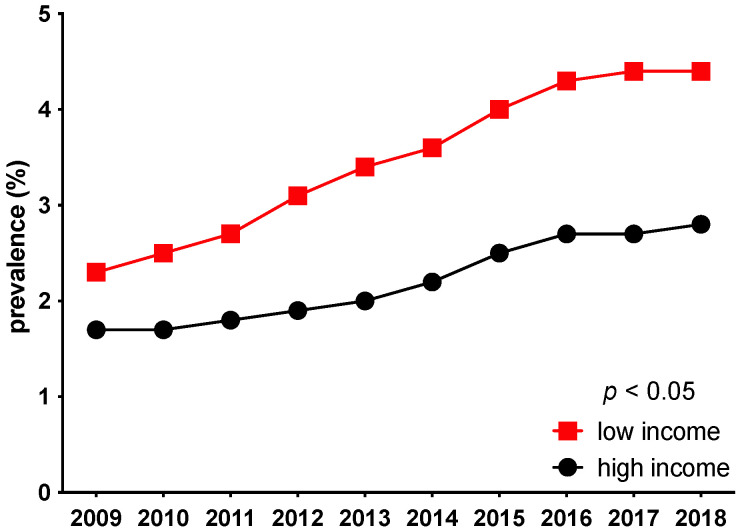
The prevalence of PAD increased during the 10-year study period, with a continuous higher prevalence in patients living in low-income areas compared with patients from high-income areas (*p* < 0.05).

**Figure 3:**
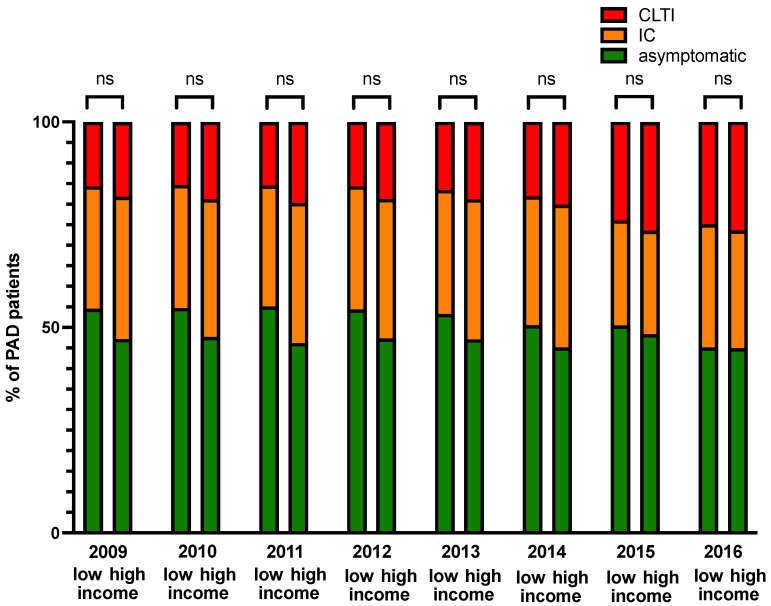
Peripheral artery disease stratified according to severity (asymptomatic, intermittent claudication (IC), critical limb-threatening ischemia (CLTI)). Severity of PAD did not differ between low-income and high-income areas (ns denotes not significant).

The prevalence of PAD increased during the 10-year study period, with a continuous higher prevalence in patients living in low-income areas compared with patients from high-income areas (2009: low-income prevalence 2.3%, high-income prevalence 1.7%; 2018: low-income prevalence 4.8%, high-income prevalence 2.8%; *p* < 0.05) (Figure 1 and Figure 2). The severity of PAD did not differ between low-income and high-income areas between 2009 and 2016 (Figure 3).

**Figure 4:**
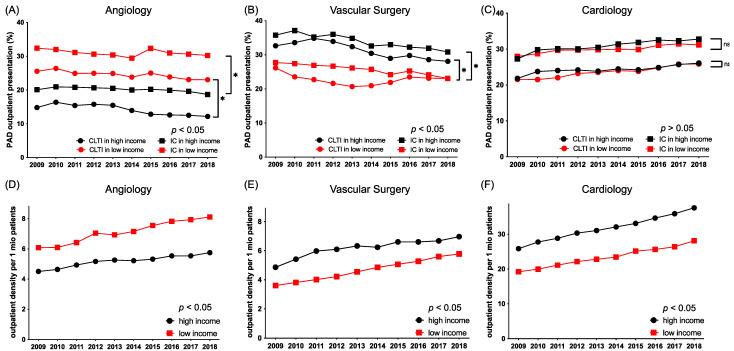
Patient presentation rates (**A**–**C**) and outpatient density (**D**–**F**) of specialized vascular care stratified by income level and specialty (angiology (**A**,**D**), vascular surgery (**B**,**E**), cardiology (**C**,**F**)) (* denotes *p* < 0.05, ns denotes not significant).

High-income areas had higher outpatient vascular surgery and cardiology density during the study period, whereas the outpatient angiology density was higher in low-income areas (Figure 4).

Although most patients had contact with a primary care physician (on average 99.8% from 2009 to 2018), only a minority presented to a vascular specialist, with 26% of patients from low-income areas and 24.9% of patients from high-income areas.

In 2009, 29% of low-income patients presented to angiology outpatient care compared to only 17.4% of high-income patients, whereas in 2018 26.6% of low-income patients presented to angiology outpatient care compared to 15.4% of high-income patients (*p* for intergroup < 0.05). Presentation rates of patients from high-income areas with more advanced stages of PAD (CLTI) to angiology outpatient care were even lower (2009 CLTI in low-income patients 25.6%, in high-income patients 14.8%; 2018 CLTI in low-income patients 23.04%, in high-income patients 12.17%) (Figure 4).

Regarding vascular surgery outpatient care, our data revealed an inversed picture. Patients from high-income areas were more likely to present to vascular surgery (2009: 34.21%, 2018: 29.5%) than patients from low-income areas (2009: 27%, 2018: 23.1%); while the gap narrowed over the years, this effect remained statistically significant (*p* < 0.05). Overall presentation rates to cardiology outpatient care were similarly low, but intriguingly were not affected by income structure (Figure 4).

**Figure 5:**
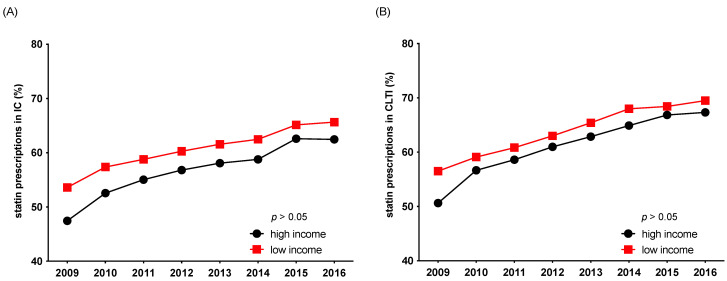
Adherence to guideline-recommended statin pharmacotherapy between 2009 and 2016, stratified by income level and stage of disease: intermittent claudication (IC) (**A**), critical limb-threatening ischemia (CLTI) (**B**).

**Figure 6:**
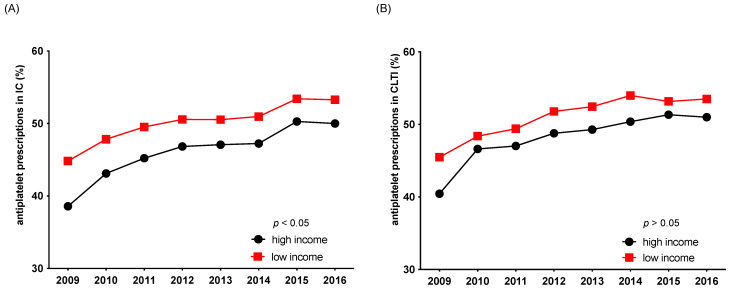
Adherence to guideline-recommended antiplatelet pharmacotherapy between 2009 and 2016, stratified by income level and stage of disease: intermittent claudication (IC) (**A**), critical limb-threatening ischemia (CLTI) (**B**).

Overall adherence to guideline-recommended pharmacotherapy (antiplatelet therapy, statins) remained low during the observed timeframe between 2009 and 2016. Patients from low-income areas received a statin in only 55% of cases in 2009 and 67.6% in 2016, whereas patients from high-income areas received a statin in 49% of cases in 2009 and 64.9% in 2016. The same pattern was shown for antiplatelet treatment (Figure 5 and Figure 6). Interestingly, patients with advanced stages of PAD from high-income areas were even less likely to receive the guideline-recommended pharmacotherapy of a statin and antiplatelet therapy than patients from low-income areas. Although this income-related gap narrowed over the observed timeframe it remained statistically significant in the case of antiplatelet prescription in patients with IC (*p* < 0.05) (Figure 5 and Figure 6).

## 4. Discussion

Atherosclerotic cardiovascular diseases are the leading causes of mortality and morbidity in the industrialized world, with substantial associated healthcare costs [30].

Studies have shown a well-established link between income and health, pointing out that people with high income are less likely to live unhealthily [31]. A Spanish study showed that preventing income disparities is an effective way to reduce health inequality [32]. Especially for cardiovascular diseases, income levels are strong predictors of cardiovascular health [2,25,33]. Subsequently, it was shown that the risk of lower extremity amputation is higher in metropolitan regions with a lower socioeconomic status [34,35]. Socioeconomic factors and education, as well as area-level socioeconomic indicators, are independent contributions to PAD onset [36].

Our study now shows that income is an important but non-linear factor potentially associated with presentation to vascular specialists and regarding prescription of guideline-recommended medical therapy. Our data highlight that patients from high-income states in Germany are more likely to present to outpatient vascular surgery than outpatient angiology care, but are less likely to receive guideline-recommended medical therapy compared with patients from low-income states.

While we repeatedly show an overall low adherence to guideline-recommended pharmacotherapy irrespective of income status, our data now suggest an interesting relation between vascular specialists and adherence to medical guideline therapy. Interestingly, areas with higher outpatient angiology density were associated with more prescriptions of guideline-recommended pharmacotherapy. Consistently, patients with progressive PAD stages in low-income areas were more likely than patients in high-income areas to present to outpatient angiology and to receive guideline-recommended therapy. While we show that the severity of PAD was not influenced by income structures and low-income individuals seem to be more likely to receive guideline-recommended pharmacotherapy, it has been previously demonstrated that mortality is associated with income status [37]. However, there are more aspects to be considered here beside pharmacotherapy. Despite being more likely to receive guideline-recommended pharmacotherapy, low-income individuals tend to have more comorbidities and cardiovascular risk factors, such as unhealthy dietary patterns or tobacco use [38,39]. A limitation, however, is that we cannot present patient-level or physician-level data; further analysis regarding prescriptions and vascular-specialist-specific treatment should be investigated in forthcoming studies. Due to the study-design interpretation of the claims data obtained, it is possible only to draw correlations, not direct causalities. Despite the lack of inpatient information, the large amount of data, comprising nearly 70.1 million insured patients per year, allows for a representative overview of outpatient-healthcare structures, with special regards to income-related inequalities in Germany. A limitation of the study is that outpatient encounters were not traced individually. Due to the retrospective design of the study, some data might be inadequate. Additionally, it was not possible to account for treatment adherence to the prescribed pharmacotherapy. Others have shown that patients form low-income areas are more prone to not comply with pharmacotherapy than patients from high-income areas [40]. Furthermore, no insight into laboratory measurements of lipid blood levels can be given. Low statin prescription rates could derive from a priori low lipid levels due to dietary measures or physical activity. Given that in most cases in clinical routine the recommended LDL-C levels are rarely met, this scenario seems unlikely [41,42]. With regards to the low frequency of antiplatelet and statin therapy, this study did not account for potential intolerances or contraindications such as bleeding complications.

In conclusion, at the regional level of the 17 associations of statutory health insurance physicians, we observed an income-related inequality in guideline-recommended pharmacotherapy and, additionally, income-related differences in treatment patterns by vascular specialists within a country with a progressive European healthcare system. Our data unexpectedly indicate that patients with PAD from high-income areas experience greater insufficiencies in treatment compared with patients from low-income areas. However, the reasons for this remain unclear and future studies are needed to examine this relationship, accounting for further risk factors, both at the individual and contextual level.

## Data Availability

Not applicable.
